# Eosinophilic Esophagitis in Esophageal Atresia

**DOI:** 10.3389/fped.2019.00497

**Published:** 2019-11-29

**Authors:** Usha Krishnan

**Affiliations:** ^1^Department of Pediatric Gastroenterology, Sydney Children's Hospital, Sydney, NSW, Australia; ^2^School of Women's and Children's Health, University of New South Wales, Sydney, NSW, Australia

**Keywords:** eosinophilic esophagitis (EoE), esophageal atresia (EA), stricture, gastroesophageal reflux, pediatrics

## Abstract

Recent studies have reported a higher prevalence of eosinophilic esophagitis in children with esophageal atresia. Under recognition of eosinophilic esophagitis in these patients may lead to excessive use of antireflux therapy and an escalation of interventions, including fundoplication, as symptoms may be attributed to gastroesophageal reflux disease. In addition, long-term untreated eosinophilic esophagitis may lead to recurrent strictures due to transmural esophageal inflammation, necessitating repeated dilatations. Eosinophilic esophagitis should be considered when children with esophageal atresia show persistent symptoms on standard antireflux treatment, increasing dysphagia, and recurrent strictures. Treatment has been found to not only significantly reduce intraepithelial eosinophil count, but also to improve symptoms, and to lower the occurrence of strictures and the need for dilatations. Future prospective studies are warranted in this area.

## Introduction

**Figure 1:**
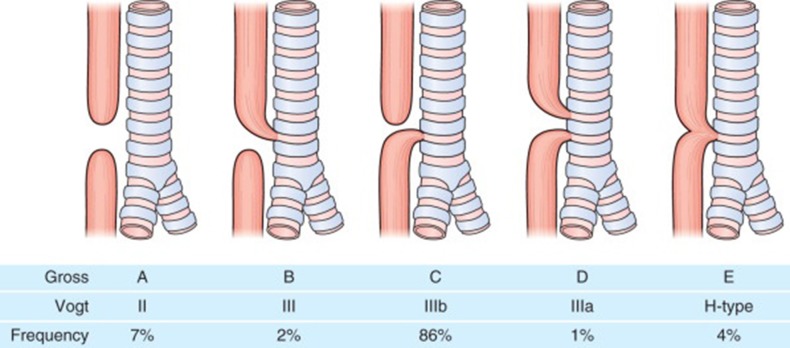
Classification of different types of Esophageal Atresia.

Eosophageal atresia (EA) is one of the common congenital gastrointestinal anomalies with an incidence of around 1 in 2500 live births. In esophageal atresia there is almost always a disruption in the continuity of the esophagus resulting in a proximal and distal esophageal pouch and depending on whether there is a communication (tracheoesophageal fistula) between the proximal or distal esophageal pouches and the trachea, the EA is classified into Types A to E (Figure 1). EA is repaired soon after birth although in cases where a long gap exists between the proximal and distal esophageal pouches a delayed repair might be done. EA carries a lifelong gastrointestinal morbidity. All patients have a degree of esophageal dysmotility, shortened esophagus and sometimes hiatal hernia which makes them prone to gastroesophageal reflux disease (GERD) for which they are sometimes fundoplicated. Apart from esophageal dysmotility these patients also have anastomotic strictures resulting in dysphagia and feeding difficulties (1).

At least 15 eosinophils/HPF on esophageal biopsy are needed for eosinophilic esophagitis (EoE) to be diagnosed. The symptoms in EoE are related to eosphageal dysfunction secondary to eosinophilic infiltration in the esophagus resulting in dysmotility and strictures.

Although recently in literature, a higher prevalence of EoE has been reported in children with EA, there are currently not many studies in published literature which look at the interplay between both these diseases. This is not only because EA is a rare disease but also because the diagnosis of EoE in EA patients can be difficult due to similar symptoms occurring due to gastroesophageal reflux disease (GERD) as well as those due to dysphagia secondary to esophageal dysmotility. Hence often either the EoE is mis-diagnosed as refractory GERD or there is a delayed diagnosis of EoE. This delay might not only result in the EA patient with EoE having unnecessary escalating therapeutic interventions for their presumed poorly treated refractory GERD but might also put them at risk for developing complications from their untreated EoE.

The prevalence, etiology, pathogenesis, symptoms, investigation and treatment of EoE in children with EA is described in this review article.

## Prevalence

EoE has been described as a separate entity only since the mid1990s, with an increasing prevalence of up to 27 per 100,000 in developed countries (2). No controlled studies on the prevalence of EoE in EA patients have been carried out. Although by 2015, 48 cases of EoE in EA had been reported, by l the beginning of 2019, this had increased to 101 reported cases of EoE in EA patients in literature (3, 4). The largest reported cohort of EoE in EA patients was in a study by Dhaliwal et al. from 2013, where a retrospective review of all biopsies from an esophageal atresia cohort of 103 showed an incidence of 17% (5). In this study there was a 364-fold enrichment of EoE in patients with EA compared to the general pediatric population with a reported incidence of EoE of 1 in 10,000 (5).

Other case series of EoE in EA patients include Yasuda et al. (15.2%), Gorter et al. (1.25%), Pedersen et al. (10%), Batres et al. (3%), Oliveira et al. (4%), Yamada et al. (6%), Kassabian et al. (5%), and Lardenois et al. (9.5%) (3, 6–13). These figures are significantly higher than the reported incidence of 8–10% EoE in children with suspected GERD not responding to standard anti-reflux treatment (14, 15).

## Etiology and Pathogenesis

Eosinophilic esophagitis is characterized by destructive responses of the immune system to environmental allergens, including food, on the human esophagus. EoE is now reported as a major cause of upper gastrointestinal morbidity in children and adults and the incidence is reported to be on the increase. One of the reasons for the increased incidence was thought to be due to aeroallergens which have been implicated in the pathogenesis of EoE. However, an adult study looking at EoE patients by Elias et al. did not find an increase in esophageal eosinophilia in the summer months, despite 69% of the patients having reactions to >4 aeroallergens in the study (16).

### Genetic Causes and Heritability

It is known that EoE has a high degree of heritability, with a majority of the phenotypic variation believed to be genetic in origin as shown by genetic epidemiology studies of twins and families. A study by Alexander et al. showed the EoE relative risk ratio to be increased 10- to 64-fold depending on the family relationship, compared with the general population. In this study, EoE in relatives was 1.8 to 2.4%, depending on relationship, and sex and twins cohort analysis revealed a powerful role for common environment (81.0%) compared with additive genetic heritability (14.5%) (17). Since 2010, three GWAS have been published identifying c11orf30, STAT6, ANKRD27, CAPN14 loci which influence risk for EoE in both children and adults (18).

### Early Life Factors

Jensen et al. have also shown a positive association between several early-life factors and EoE, including prenatal (maternal fever: adjusted odds ratio [aOR], 3.18; 95% CI, 1.27–7.98; preterm labor: aOR, 2.18; 95% CI, 1.06–4.48), intrapartum (cesarean delivery: aOR, 1.77; 95% CI, 1.01, 3.09), and infancy (antibiotic use: aOR, 2.30; 95% CI, 1.21–4.38; use of an acid suppressant: aOR, 6.05; 95% CI, 2.55–14.40) factors (19, 20). A previous study by the same author had shown an even higher risk (6 times) between antibiotic use in infancy and odds of having EoE (95% CI 1.7–20.8) (21). A more recent study by Jensen et al., found an association between genes (CAPN14, 5q, 11q,12q,2p, and LOC283710/KLF13) and early-life environment factors (breast-feeding and NICU admission), which could potentially contribute to EoE susceptibility. They found that breast-feeding in those with the susceptibility gene variant (CAPN14) reduced the risk of EoE (adjusted odds ratio, 0.08; 95% CI, 0.01–0.59) (22).

### Acid Suppression

Apart from Jensen, other studies have also postulated that early and prolonged exposure to acid suppression medication may trigger IgE-mediated food allergies in EA patients. Untersmayr et al. found that reduction in gastric acid increased the allergenicity of food proteins (23). This hypothesis has also been recently supported in mouse models where PPI use can cause the formation of food-specific immunoglobulin (Ig) E antibodies and trigger food allergy (24, 25). Orel et al. have also described other possible mechanisms by which PPI exposure might potentially lead to an increased risk of development of EoE in patients, due to their adverse influence on mucosal barrier function, interference with pH-related protein digestion by pepsin, and antigen processing by immune cells (26). Orel et al. stated that, acid suppressive medications may interfere with peptic food digestion, thereby contributing to an increase in food-specific antigens, and may also increase mucosal permeability. These effects together may cause increased allergic reactivity to foods over time, perhaps sensitizing persons and eventually triggering EoE (26). This is especially of importance in EA patients who are exposed to PPIs from birth for prolonged periods of time. This early reduction in gastric acid could potentially increase the allergic reactivity to foods by reduced peptic digestion of food proteins and increased mucosal permeability of food proteins and thereby increase the risk for development of EoE.

EA patients are also often premature and delivered by cesarean section especially if the EA is diagnosed antenatally and associated with cardiac and other anomalies. EA cohort are all also exposed to antibiotics post EA repair and for treatment of their recurrent chest infections in early life. EA cohort who are routinely admitted to NICU post EA repair after birth, are often formula fed due to feeding difficulties. As antibiotic use and early exposure to acid suppression cannot be altered in the EA cohort, persisting with breast feeding in early life and reducing the duration of exposure to acid suppressive medication would potentially be of added benefit in this cohort, although these assumptions would need to be validated in prospective long term studies. Thus, there could be several early life factors in the EA cohort which could potentially increase their risk for subsequent development of EoE. Some of these early life factors are potentially modifiable and hence if confirmed would have implications for improved understanding of EoE pathogenesis and disease prevention, in the EA cohort.

It is now accepted that EoE is the result of a T-helper cell 2–type immune response in which eotaxin 3 and interleukins (IL) 4, 5, and 12 and 13 are upregulated (27–29). The gene for eotaxin-3, which is a chemoattractant and activating factor for eosinophils, has been shown to be increased 53-fold above normal levels in patients with EoE (28, 30, 31). Both EA and EoE are polygenic conditions, and recently, Gorter et al. postulated a possible genetic association between EA and EoE through mutations in the FOX gene cluster (6). In humans, the FOX gene cluster has been shown to be associated with congenital malformations in the esophagus and lung including EA and also with binding sites for FOXF1 were also found in the promoter regions of genes for eotaxin-3 and IL-8 (6, 27, 30–34). Recently, the transcriptomes of EA patients with and without EoE, patients with EoE but without EA and healthy controls were compared, showing approximately 25% of EoE signature genes, resulting in abnormal epithelial barrier and type 2 immune-associated gene expression were dysregulated in those born with EA (at baseline) but without EoE compared to healthy controls; these genes were also found to be even more dysregulated in those with EoE but without EA and in EA patients with EoE. The dysregulated genes included genes related to esophageal epithelial type 2 inflammation (MUC4, a specific mucin in response to TH2 cytokines; SYNPO2, a gene upregulated in EoE mucosa; and FLG, a membranal barrier molecule downregulated in patients with EoE).The presence of this genetic dysregulation in patients born with EA at baseline before the development of EoE might be the reason why there is a higher prevalence of EoE in this population. Prospective longitudinal studies are needed to confirm whether these patients with EA but with dysregulated EoE-predisposing genes at baseline would develop EoE in the future. Large prospective longitudinal studies would also be helpful in determining whether prolonging breast feeding and reducing duration and cumulative dosage of exposure to proton pump inhibitor (PPI) therapy in EA patients with EoE susceptible genes would reduce their relative risk of developing EoE in the future. Interestingly, EA patients with EoE and EoE patients without EA had similar molecular transcriptomes at time of diagnosis of EoE and in remission after treatment, which is likely to be due to a similar pathogenesis induced by food allergy. In this study there was similarity in the predominance of Caucasian race, male gender and food allergy status of both the EA and non EA group with EoE, supporting that EoE in the EA cohort is the same disease as the conventional EoE in the general pediatric population. However, children with EA developed EoE at a younger age than those with EoE alone. This underscores the importance of being aware of the risk of EoE and performing endoscopies with sufficient numbers of biopsies at multiple levels, irrespective of the age in symptomatic EA patients. Also EA patients with EoE had a more-severe clinical phenotype, with higher incidence of dysphagia, episodes of food bolus impaction and strictures requiring dilation than in those with EoE alone without EA and EA patients without EoE, highlighting the importance of timely diagnosis and treatment of EoE in EA patients (31). This study found that 2 genes (ANO1 and CTNNAL1) were expressed more in EA patients with EoE than in EoE patients without EA. ANO1 is expressed by the interstitial cells of Cajal and is a calcium-activated chloride channel governing gastrointestinal smooth muscle contraction rhythms, which might be associated with dysphagia, food bolus impaction, and stricture development phenotypes observed in patients with EA and EoE. In addition, ANO1, is also an esophageal cancer marker, which is especially important in EA patients who are susceptible to esophageal squamous cell carcinoma development (31). Thus, ANO1 could potentially be used as a molecular marker for a malignant EA prognosis, EoE symptom monitoring, and esophageal cancer prognosis.

### GERD

Apart from a possible genetic, molecular association, and early life factors, other hypotheses have also been postulated explaining the increased incidence of EoE in EA patients. In EA after restoration of the esophageal interruption by surgery in the neonatal period, the esophageal dysmotility and predisposition to severe GERD persists. Hence as EoE an allergic insult on mucosal epithelium by food antigens or aeroallergens plays a role (35).

Acid peptic mucosal injury due to severe GERD may impair the mucosal barrier function in EA patients and thereby increase the risk of sensitization to food and aero-allergens thereby increasing the risk of developing EoE. It has been shown that the normally impermeable esophageal mucosa when exposed to acid becomes permeable for peptides up to 20 KD thereby allowing food allergens to enter the sub-epithelial layer and induce eosinophilic inflammation (36, 37).

### Esophageal Dysmotility

Esophageal dysmotility is almost universal after EA repair and is mainly related to the developmental anomaly and abnormal neural innervation of the esophagus. Additional post-natal causes which can worsen this dysmotility include damage to the vagus during surgical repair of the EA, GERD, EoE, strictures and post fundoplication. Currently, the management of esophageal dysmotility in patients with EA is essentially based on treatment of associated inflammation related to peptic or eosinophilic esophagitis. Esophageal dysmotility is involved in the pathophysiology of numerous symptoms and comorbidities associated with EA such as GERD, EoE, aspiration and respiratory complications, and symptoms of dysphagia and feeding disorders (38). Esophageal dysmotility and prolonged bolus clearance time (**Figure 9**) in EA patients could also result in increased exposure to potential allergens in the esophageal mucosa which is already inflamed due to acid reflux. This sustained exposure to potential food and aero allergens resulting in sensitization could result in a T-helper cell 2 immune response (6). Food impactions secondary to anastomotic strictures in EA patients might also result in mucosal damage, facilitating esophageal eosinophil infiltration (10).

### Long Gap EA

There was a higher incidence of EoE in LGEA patients in Dhaliwal et al.'s study (5). A possible explanation why LGEA patients had the highest incidence of EoE could be because these patients have more severe abnormalities to neural innervation of the esophagus at birth, suffer further damage to the vagi at time of delayed repair, have the most severe reflux and are often on high dose PPI therapy to control their severe GERD, higher rate of recurrent strictures needing dilations and food bolus impactions, and of all the types of EA LGEA have the most severe esophageal dysmotility (often aperistaltic), all of which are potentially predisposing factors for the development of EoE due to increased and prolonged exposure of the esophageal submucosa to potential allergens (Figure 9).

These antenatal, post-natal, early life environmental and other factors suggest that the increased incidence of EoE is more likely to be secondary to causality rather than coincidence (Table 1).

**Table 1:** Factors predisposing EA patients for the development of EOE.

Early life factors
Prematurity
Cesarean section
NICU admission
Early exposure to antibiotics
Early exposure to acid suppression
Genetic
Mutation in FOX gene cluster affecting Eotaxin 3 and IL 8
Abnormality in EoE signature gene even at baseline prior to development of EoE in EA patients -Gene affecting epithelial barrier function (FLG) Abnormality in esophageal epithelial type 2 inflammation associated gene MUC4 and SYNPO2
Increased expression of gene ANO1 and CTNNAL1 which are present in interstitial cells of cajal and thought to be linked to symptoms of dysphagia, food bolus impactions and strictures
Factors affecting mucosal barrier and resulting in esophageal epithelial injury
GERD
Esophageal dysmotility
Food impactions
Recurrent anastomotic strictures requiring dilation
Other Factors
Exposure to antibiotics due to recurrent chest infections
Prolonged exposure to acid suppression due to GERD
Sensitization to food and aero allergens at a rate similar to EOE patients without EA

### Strictures

The etiology of strictures in EA patients is multifactorial. Early strictures in EA patients are thought to be due to tension and relative ischemia at the site of anastomosis. Late strictures are thought to be secondary to GERD (39, 40). Strictures in EoE patients occur due to the eosinophilic inflammation affecting all three layers of the esophageal wall, and can short or long (8). Conventional treatment for EoE strictures is dilation especially in adults. However, as topical corticosteroids can reverse the sub-epithelial fibrotic process, it might be worthwhile trying topical budesonide slurry initially especially if the stricture is not too tight and the dysphagia is not severe (41). In Dhaliwal's study two EoE patients with a proximal stricture had endoscopic and symptomatic response to topical corticosteroids (**Figure 3B**), without need for stricture dilation, which supports the inflammatory nature of the stricture of EoE (5).

## Clinical Presentation

### Age of Presentation

In literature, most of the study subjects with EA and EoE are children and adolescents with ages ranging from 8 months to 17 years. The median age for diagnosis of EoE in EA patients was younger in Dhaliwal et al. (18 months), whilst it was higher in Oliveira et al. (9.8 years), Gorter et al. (12.5 years), and Kassabian et al. (6.7 years) (5, 6, 8–11). Although there was an overall male 13:7 predominance in the studies of Batres et al., Yamada et al., Gorter et al., Oliveira et al., and Kassabian et al., put together, in Dhaliwal's larger study there was a slight female predominance of 1.6:1 (5, 6, 8–11).

### Long Gap EA

Although the majority of patients with EA and EoE in the study of Dhaliwal et al. had Gross type C EA (94%) 28% had long gap (LG) EA And LGEA patients had an 11.8 times relative risk for developing EoE in this study (5).

### Vomiting and Dysphagia

With regard to symptoms at time of presentation, in the study of Dhaliwal et al., the incidence of vomiting (*p* < 0.0001) and dysphagia (*p* < 0.0001), in EoE patients was significantly higher than that in the other EA patients (5). Vomiting was also reported in 67% in Oliveira's study and in 50% in both Batres's and Yamada's study (8–10). The finding of vomiting in the EoE group could be explained by the considerable overlap between EoE and GERD symptoms in the EA cohort, and also because the esophageal dysmotility due to EoE can potentially exacerbate GERD. EoE patients in the study of Dhaliwal et al. also underwent significantly more fundoplication when compared with those without EoE, *p* < 0.0.0001, which could have been due to EoE being mis-diagnosed as refractory GERD (5). The importance of possible misdiagnosis of EoE as GERD was also highlighted in a study by Pesce et al. where nearly 1 in 4 patients, including those in the EA with EoE group, had already undergone an anti-reflux surgery at time of diagnosis of EoE at baseline (42). In the same study by Pence et al., they did not find any symptoms which could distinguish between EA patients with EoE from EA patients without EoE or GERD patients, highlighting not only the difficulty of diagnosing EoE based on symptoms alone but also the importance of endoscopy and biopsy for diagnosis of EoE, in the EA cohort, especially in those being considered for fundoplication (42). In a prospective study on 63 adolescents with EA by Lardenois et al. chest pain was the only symptom to occur significantly more in EA patients with EoE compared to EA patients without EoE (12).

### Feeding Difficulties

The incidence of gastrostomy was also greater in the in EA patients with EoE (33%) than in EA patients without EoE (13%) in Dhaliwal et al. study (5). EA patients with GERD, dysphagia, and feeding difficulties often require a gastrostomy for supplemental feeds, and treating their EoE in addition to their GERD may potentially reduce the need for naso-gastric feeds and placement of gastrostomy. However, long term follow up outcome studies post treatment of EoE are needed in a larger cohort to support this hypothesis.

### “Hypoxic/cyanotic Spells”

Interestingly, in Dhaliwal et al. study EoE patients also had a significantly higher incidence of “hypoxic/cyanotic spells” (*p* = 0.03) (5). The etiology of “hypoxic spells” in EA patients is multifactorial and thought to be secondary to tracheomalacia, GERD, esophageal dysmotility, and strictures. The authors in this study postulated that the higher incidence of “hypoxic spells” in EA patients with EoE in this study could potentially be due to worse esophageal dysfunction and stricture rate in the EA with EoE cohort (5). The severe dysmotility and increased stricture rate in the EA with EoE cohort could lead to food bolus impactions resulting in ballooning of the esophageal pouch proximal to the anastomotic site during feeding, causing tracheal occlusion and severe hypoxia otherwise known as, “hypoxic/cyanotic spells.” This highlights the importance of excluding not only tracheomalacia and GERD but also EoE in EA patients with “hypoxic spells,” especially in the presence of severe dysphagia with or without stricture. This finding however, needs to be confirmed in larger prospective studies investigating the etiology of “hypoxic spells” in EA patients.

### Strictures

**Figure 2:**
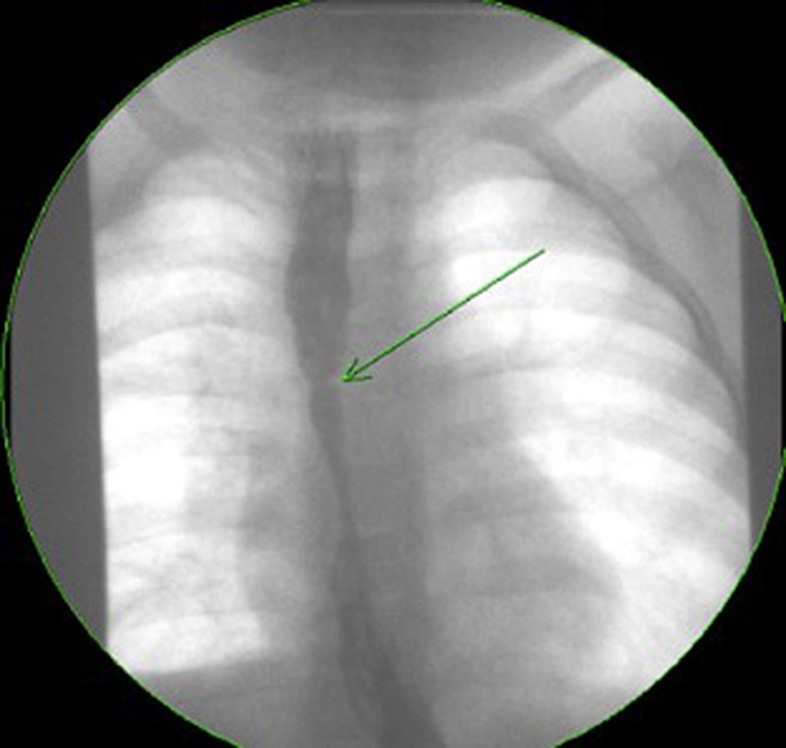
Contrast study of a symptomatic esophageal atresia (EA) patient with an eosinophilic esophagitis (EoE) stricture.

**Figure 3:**
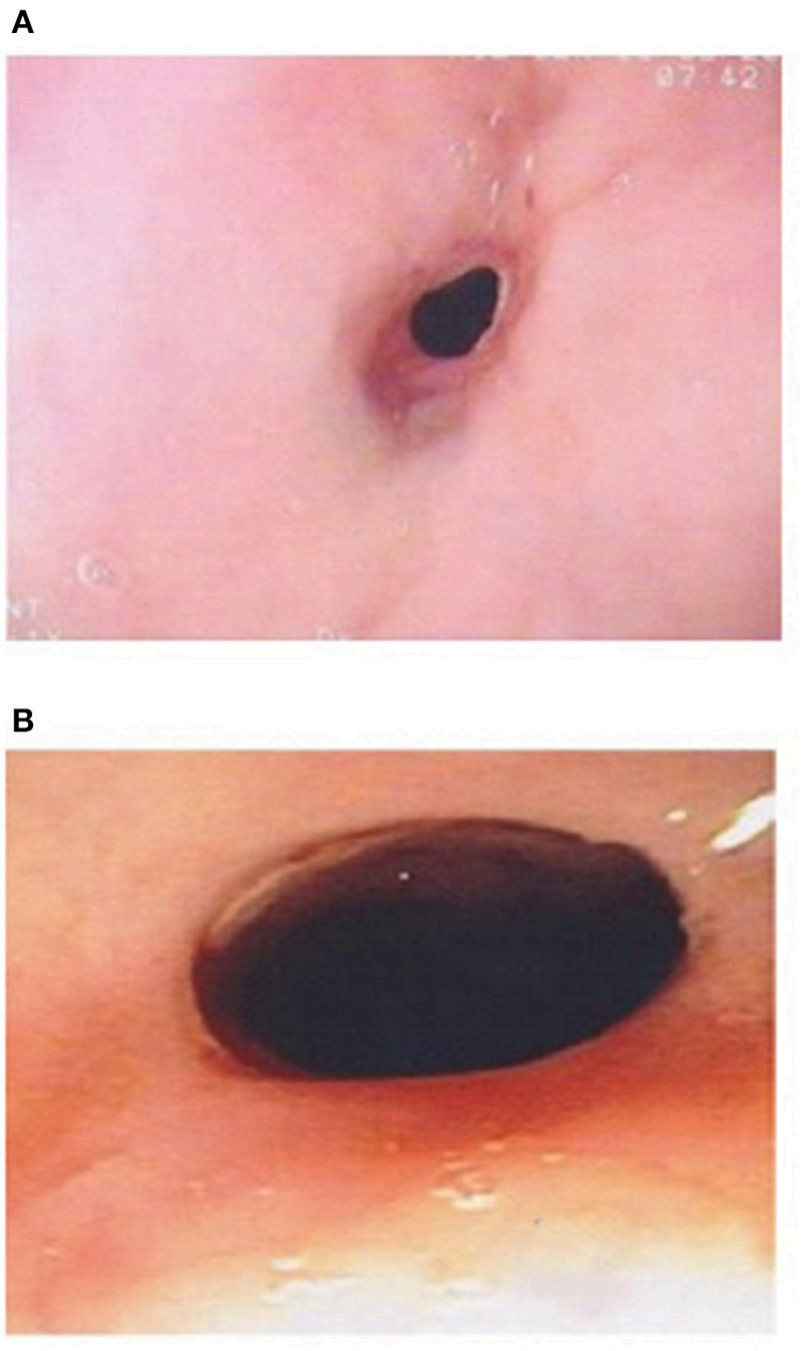
**(A)** Esophageal stricture in esophageal atresia (EA) patient with eosinophilic esophagitis (EoE) pre medical treatment of EoE. **(B)** Endoscopic improvement of esophageal stricture in esophageal atresia (EA) patient with eosinophilic esophagitis (EoE) post medical treatment of EoE.

Esophageal strictures occur in 5–15% of cases of EA, often in the first year of life (8, 39). In the study by Pesce et al. the age at diagnosis of strictures did not differ between the EA groups with and without EoE (42). Figure 2 shows a contrast study in an EA patient with a stricture secondary to EoE, which was subsequently diagnosed on endoscopy (Figure 3A) with biopsy of the stricture site. Strictures were reported in 20% in Kassabian's study, 50% in Oliveira's study, 100% in Batres's study and 83% in Yamada's study (8–11). In Pesce et al. study the presence of esophageal mucosal eosinophilia was the most predictive factor for stricture formation in EA patients. In Dhaliwal's study 38% had a stricture at time of diagnosis of EoE, and a significantly greater number of EA patients with EoE developed late strictures (>1 year of age) when compared with those without EoE (5). In this study EA patients had a 1.9 times relative risk for stricture formation if they had EoE, long gap, or both (5). The likelihood of long gap EA patients with EoE developing strictures was 4:1 (5).

**Figure 4:**
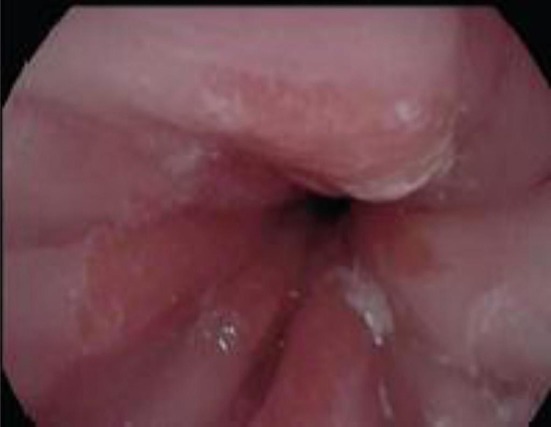
Endoscopy showing abnormal salmon colored mucosa arising from the lower esophageal sphincter.

**Figure 5:**
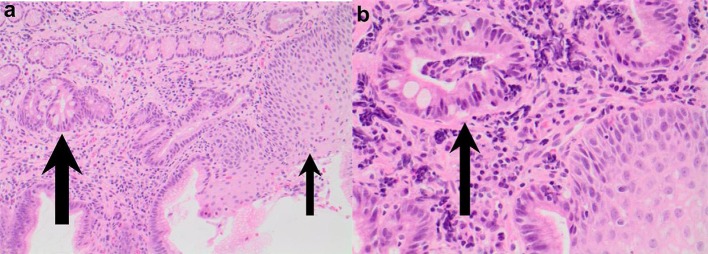
**(a,b)** Esophageal biopsies from salmon colored mucosa showing eosinophilic infiltration of squamous mucosa (thin arrow) and intestinal metaplasia within glandular mucosa (thick arrow; H&E 200) and intestinal metaplasia confirmed by presence of goblet cells (thick arrow) in biopsy taken from abnormal colored mucosa (H&E 400).

Of concern, a case report by Tan et al. described a 4 year old girl with EoE and concomitant Barrett's changes with intestinal metaplasia post EA repair (Figures 4, 5a,b). This patient is the youngest EA patient in reported literature with intestinal metaplasia. She remains the first and only such reported patient with both EoE and Barrett's esophagus post EA repair. It remains unclear if EoE is also an independent risk factor to GERD for the development of Barrett's esophagus (43).

### Atopy and Food Allergy

In EoE coexisting atopic conditions are extremely common, with up to 42–93% of pediatric patients having another allergic disease (14). In Dhaliwal's study, 50% had a coexisting atopic condition, and EA patients with EoE also had a significantly higher incidence of reactive airway disease, *p* < 0.0001. The 55% incidence of reactive airway disease in this study in the EA with EoE cohort was comparable with that reported in literature in EoE patients without EA (44). Atopy was also common in Oliveira (87.5%), Batres's (66%), Gorter's (50%), and Lardenoise study (6, 8, 12). The FIGERS consortium found that 67% of children with EoE had a positive skin prick testing (SPT) to at least one food (14). Spergel et al. found that milk, egg, wheat, and soy were the most common foods causing EoE (45). Coexisting food allergies were reported in in 67% in Yamada's and Batres's and in Oliveira (75%), Gorter (100%), and Chan (40%) studies (6, 8–10, 44). The EoE patients identified in Dhaliwal's and Chan's studies too had positive SPT to diary, egg, and peanut (5, 44).

### Quality of Life

EA patients with EoE can have a low quality of life (QOL). The authors' own data from a EA multidisciplinary clinic showed that EA patients with EoE scored lower total scores in in a generic, pediatric QOL questionnaire (PedsQL-EoE module) compared to EA or EoE only groups (Poster at EA conference Rotterdam 2014).

## Investigations

### Endoscopy and Biopsy

**Figure 6:**
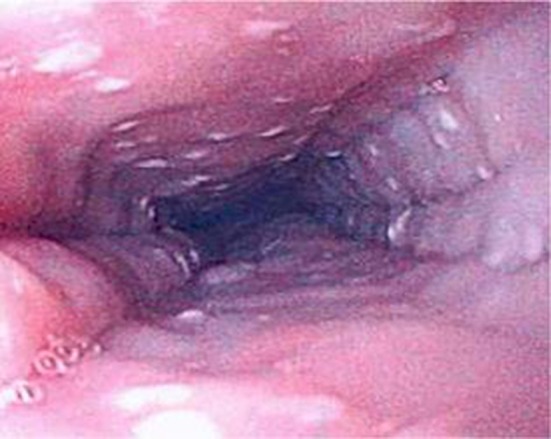
Endoscopic appearance of esophageal atresia (EA) patient with eosinophilic esophagitis (EoE) showing furrowing and white exudate.

EoE can only be definitively diagnosed with an endoscopy and biopsy which shows >15 eosinophils/HPF (14). As EoE is a patchy disease process it is important to take biopsies from multiple levels in the esophagus (46). Furthermore, the typical endoscopic findings of EoE, namely furrows, circular rings and, white exudates (Figure 6), may not be seen in all patients. In Dhaliwal's study furrows, white plaques were seen in 56%, In Oliveira's study furrows were seen in 75% and white plaques in 50%, but these were not seen in Yamada's and Batres's study (5, 8–10).

### Allergy Testing

Due to the atopic nature of EoE skin prick test and atopic patch test were initially considered to be of some diagnostic value (15, 45). However, the predictive value of both tests shows great variability and nowadays they are no longer considered to be useful and empiric two, four, six and rarely 10 food elimination diets are used which are not based on results of SPT (15, 45, 47, 48). Peripheral serum eosinophilia was seen in all EoE patients in Oliveira's and Gorter's studies, but was seen in only 33% in Batres's study and 22% in Dhaliwal's study and was associated with the diagnosis of EoE in Pence et al. study (5, 6, 8, 9, 42).

### pH-Impedance Testing

A recent study by Pesce et al. on EA patients with EoE concluded that pH-impedance testing had a role in the diagnosis of EoE as higher esophageal acid exposure time (EAT) and lower baseline impedance (BI) values were significantly associated with esophageal hyper eosinophilia (42).

According to the most recent EoE guidelines, diagnostic criteria for EoE require (1) symptoms of esophageal dysfunction, (2) >15 eosinophils/HPF on esophageal biopsy, and (3) exclusion of other potential causes for symptoms and biopsy findings (49). Application of these criteria to EA patients is problematic, as many EA patients experience symptoms of esophageal dysfunction related to abnormal esophageal development, post-surgical sequelae, dysmotility, anastomotic strictures and GERD. Hence it is often impossible to exclude these factors as contributors to symptomatology, and many may meet the >15 eosinophil/HPF cut off with alternative reasons for esophagitis such as severe GERD. This suggests that use of eosinophil count alone in EA patients to diagnose EoE as previously reported is likely insufficient. Other clinical factors such as gross endoscopic findings consistent with EoE (e.g., furrowing, exudate, circular rings) or by coexistence of other atopic disorders and food allergy, or family history also have a role to play. The recent study by Krishnan et al. looking at the EoE transcriptome showed identical abnormalities in the transcriptome at baseline at time of diagnosis of EoE and on remission post treatment in EA patients with EoE and EoE patients without EA supports the hypothesis that the disease (EoE) is same in the EA cohort and the general pediatric population (31). In this study these abnormalities in EoE signature genes were not seen in controls or GERD patients (31). To avoid over diagnosis of EoE in EA patients there might be a role for evaluating the EoE transcriptome at time of diagnosis of EoE in EA patients. There is a need for adapted criteria for EoE diagnosis in the EA population to better identify candidates for EoE-directed therapies. The modified adapted criteria for diagnosis of EoE in the EA cohort could potentially include evaluation of the EoE transcriptome, pH-impedance testing and contrast study in addition to >15 eosinophils/HPF on biopsy in a symptomatic EA patient.

## Treatment

Treatment of EoE in the EA cohort is similar to the treatment of EoE in the general pediatric population. Treatment options for EoE include dietary therapy in the form of elemental or empiric elimination diets, or local or systemic steroids (14, 50). A subset of EoE patients respond well to PPI treatment. Although previously, one needed to exclude PPI responsiveness of esophageal hyper eosinophilia before making a diagnosis of EoE (51), nowadays there is ample evidence that PPIs should be part of the treatment algorithm of EoE. In studies looking at responsiveness of EoE patients to PPI, overall, histologic response ranged from 23% to 83%, and clinical responses were 23 to 82%. In a meta-analysis by Lucendo et al., the pooled histologic response to PPI treatment in children with >15 eosinophils/HPF was 54% (95% CI, 38–70). There are multiple plausible mechanisms whereby EoE patients benefit from PPI: first anti-inflammatory effects of PPIs might decrease acid injury-related pro inflammatory cytokines, and esophageal permeability. They also have a direct anti-inflammatory effect by scavenging reactive oxygen species (52–54). Second, PPIs inhibit IL-4-stimulated eotaxin-3 expression by reducing the binding of STAT6 to the exotaxin-3 promoter in esophageal cells thereby potentially reducing eosinophil recruitment (55). Third, PPIs can also exhibit antioxidant properties and inhibit certain functions of immune cells that may contribute to EoE. Fourth, PPIs can also heal damaged and inflamed esophageal mucosa due to their effect on gastric acid by reducing acid reflux and thereby potentially reduce further antigen/allergen activation. These effects of PPIs are unrelated to their gastric acid-inhibiting effects (49, 56). Looking at the effect of PPIs on EoE in the EA cohort, in the study by Yamada et al., two (33%) patients treated with esophageal dilatation followed by PPI showed both a symptomatic improvement and a reduction in the eosinophil count in the biopsy (10). In Dhaliwal's study 28% [5] had histological remission and symptomatic improvement on PPI therapy alone (5). However, often this initial reduction in eosinophil numbers in response to PPIs may not be sustained (57). Elemental diets are known to resolve clinical as well as histologic findings in 95% of cases of EoE in the general population (45). However, compliance is often low especially in older children (45). Alternatively, elimination diets (two, four, six, 10 foods) can improve symptoms resolve histologic findings in up to 75% of cases (45). Topical steroids can improve symptoms in 75% and result reduction in eosinophil numbers on histology in 50% of cases (58–60). Generally, systemic steroids resolve EoE during the period of intake; but relapse is seen in 35% after withdrawal (59).

**Figure 7:**
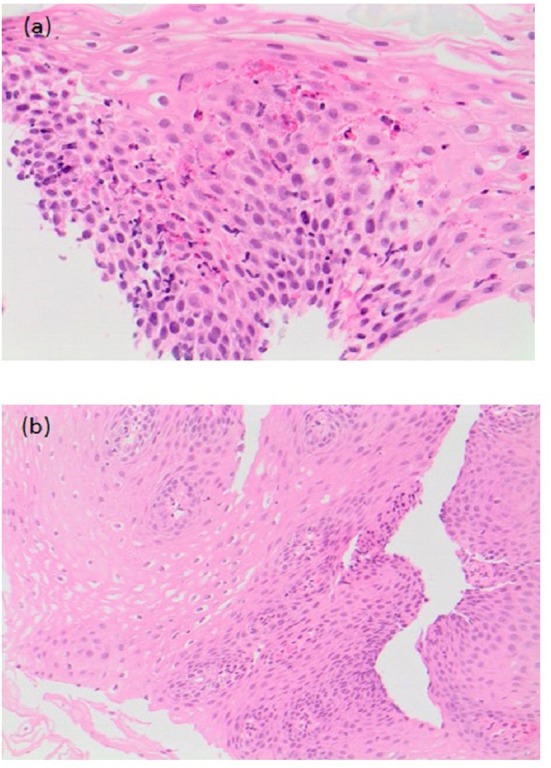
**(a)** Pre-treatment histological slide of esophageal atresia (EA) patient with eosinophilic esophagitis (EoE) showing an increase in intraepithelial eosinophils (>15/HPF). **(b)** Post-treatment slide of the same EA patient showing histological improvement with reduction in the number of intraepithelial eosinophils.

The study by Chan et al. was the first to look at the effect of treatment of EoE in 20 EA patients (44). Median age at diagnosis was 26 months (8–103 months) and median time from diagnosis to last follow-up was 23 months (2–132 months). Patients were treated with budesonide slurry, swallowed fluticasone or elimination diet alone or in combination. All patients were also on PPIs at time of diagnosis of EoE, which was continued. Furrowing/exudate seen on endoscopy at diagnosis resolved on treatment in 6 out of 7 patients at a median follow-up period of 26 months (*p* = 0.031). Median peak intraepithelial eosinophil count reduced significantly from 30/HPF (19–80/HPF) to 8/HPF (0-85/HPF) (median time for improvement 24 months; *p* = 0.015), and there was an improvement in the associated reactive changes in addition to a reduction in the peak eosinophil count on histology (Figures 7a,b) (44). There was also a significant reduction (*p* < 0.001) in symptoms of dysphagia, reflux and strictures needing dilations post treatment (44).

In a recent study by Pesce et al. the mean follow-up was 22 months. The treatment regimen was PPI with budesonide in 15/19 patients, PPIs and diet (empiric six-food elimination diet of soy, egg, milk, wheat, nuts, and seafood) in 4/19 patients. In all patients, there was symptomatic improvement and remission of EoE on histology. Furthermore, there were no new episodes of food bolus impaction and/or strictures recurrence post treatment of EoE at follow-up (42). In Dhaliwal et al.'s study, although 7 patients (38%) had an esophageal stricture at time of EoE diagnosis, only 5 were dilated at time of the initial endoscopy, and 2 patients had resolution of their strictures on medical treatment of their EoE alone and did not require further dilatation (5). In Chan et al. study, prevalence of strictures significantly decreased (*p* = 0.016), as did need for dilatations (*p* = 0.004), post EoE treatment in EA patients (44).

In the study by Yasuda et al. which also looked at treatment outcomes in 31 EA patients with EoE, 10 (32%) were PPI responders, 5 responded only when H2 receptor antagonists was added to PPI, 6 responded only to viscous budesonide and 1 to elimination diet, 1 to the addition of erythromycin as a prokinetic and 3 spontaneously improved over time. Five of the remaining non-responders are being followed up on high dose PPI therapy alone (13). This study was limited by incomplete follow up data as only 66% of EA patients diagnosed with EoE were followed up.

**Figure 8:**
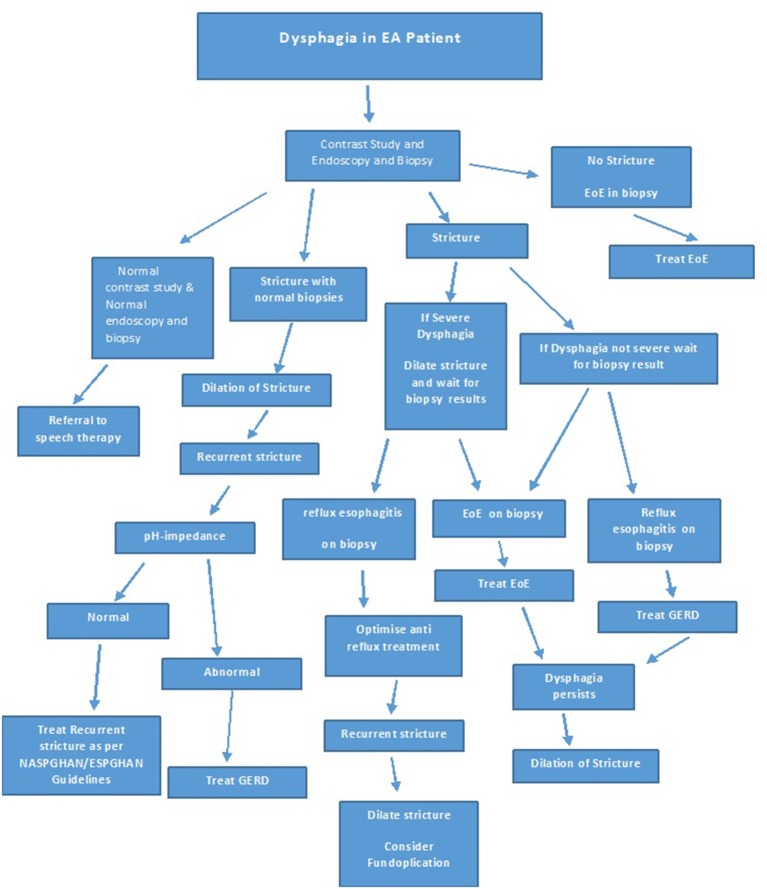
Algorithm for investigation and treatment of dysphagia in a EA patient.

The studies showing improvement in dysphagia, stricture recurrence and food bolus impaction post treatment of EoE in EA patients underline the importance of investigating new onset or increasing dysphagia in EA patients not only by a contrast study, but also with an endoscopy with biopsies at multiple levels, prior to dilatation. If EoE or GERD is identified on histology, it should be treated and dilatation should be reserved for those with refractory strictures despite optimal treatment of EoE and or GERD, or if clinically indicated. Figure 8 shows an algorithm for the investigation and treatment of an EA patient presenting with symptoms of dysphagia.

**Figure 9:**
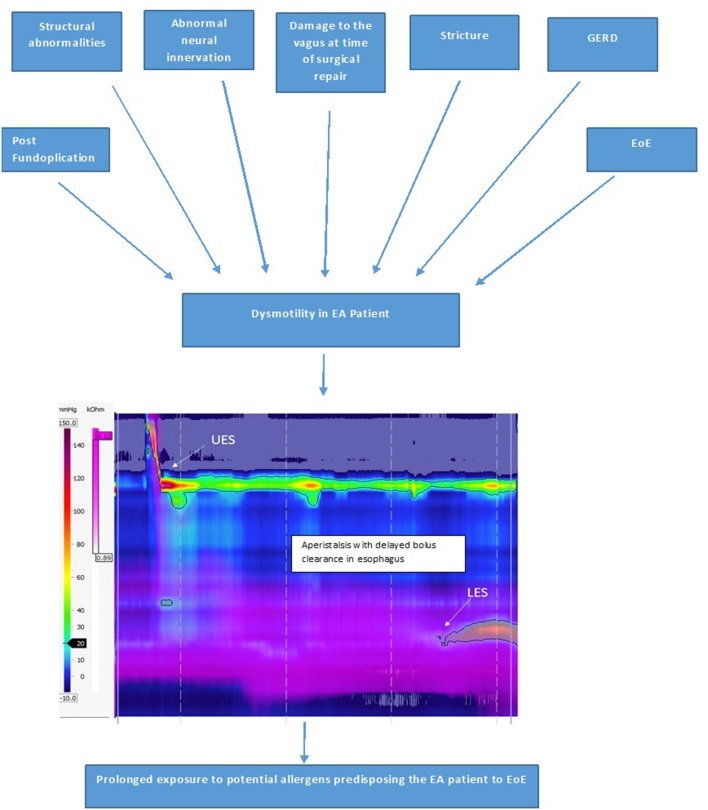
Factors causing esophageal dysmotility in the EA patient, which predisposes them to development of EoE and High-resolution impedance manometry tracing of the esophagus in a patient with type C esophageal atresia showing dysmotility with aperistalsis, incomplete bolus clearance with residual liquid in the distal esophagus.

EA patients often present with feeding difficulties and have gastrostomies to optimize nutrition. In Chan et al.'s study, feeding in 4 of the 6 patients with gastrostomies at baseline at time of diagnosis of EoE improved on treatment of EoE and the gastrostomy could be closed (44). There was also a trend toward improvement in weight and height “z scores” of all EoE patients post treatment of their EoE (44). Although these findings need to be confirmed with larger prospective studies, they highlight the importance of excluding EoE with an endoscopy and biopsy in EA patients with unexplained feeding difficulties.

## Conclusion and Future Perspective

Recent studies suggest an increased prevalence of EoE in EA patients. As presenting symptoms of EoE are similar to those of GERD, misdiagnosis or delayed diagnosis often occurs in EA patients, in whom anastomotic strictures, GERD and dysphagia are common. Due to this considerable symptom overlap the consensus guidelines on the management of gastrointestinal and nutritional complications in EA patients by ESPGHAN/NASPGHAN societies, recommended that EoE be excluded with endoscopy and multiple biopsies both proximal and distal to the anastomotic site, in EA patients of all ages with dysphagia, reflux symptoms, coughing, choking or recurrent strictures that are refractory to PPI, before proceeding to fundoplication (61). It is especially important to exclude EoE in EA patients with recurrent strictures rather than routinely dilating these strictures without further investigation as used to be the norm in the past. If EoE is diagnosed on biopsies, these children may respond to treatment of the EoE alone, with dilatation being reserved for those with refractory strictures in spite of an adequate EoE and/or GERD treatment or if clinically indicated. EA patients with recurrent strictures often proceed to fundoplication for presumed refractory GERD. It is essential to exclude EoE, before proceeding to fundoplication in EA patients as EoE may often mimic GERD in these patients. There might also be a role of excluding EoE in EA patients with unexplained feeding difficulties being considered for gastrostomy placement. Treatment of EoE in children with EA patients leads to not only mucosal healing but also results in a significant improvement in clinical symptoms, recurrence of strictures needing dilatations and episodes of food bolus impactions.

This review provides a comprehensive overview of the prevalence, etiology and pathogeneses, presentation, investigation, treatment and response to treatment of EoE in EA patients. The new data summarized for the first time in this review include data on EoE transcriptome in EA patients which predisposes them to EoE and also the data the abnormal acid exposure time in EA patients with EoE which highlights the importance also doing pH-impedance testing in these patients in addition to endoscopy and biopsies. New data is also presented on the severe phenotype and response to the various treatment modalities for EoE in the EA cohort.

It is important in the future to do prospective long-term longitudinal multicenter follow-up studies in a large cohort of EA patients with EoE, not only to determine the true incidence of EoE in this cohort, but also to look at the effect of timely detection and treatment of EoE on symptoms including dysphagia, stricture recurrence, episodes of food bolus impaction, referral for fundoplication and gastrostomy and growth and nutrition. It would also be important to determine the effect of EoE and its treatment on quality of life of these patients and their family. An ESPGHAN multicenter collaborative study (ESPGHAN Networking Grant 2017) looking at the incidence, presentation, diagnosis and treatment of EoE in EA patients which is going in commence in late 2019 and be conducted over 2 years will hopefully address some of the deficiencies in the EoE in EA literature by using validated tools for symptoms (Pediatric Eosinophilic Esophagitis Symptom Scales- PEESS), quality of life assessment (PedsQL-EoE module), endoscopic (EoE Endoscopic Reference Score-EREFS) and histologic features (EoE histologic Scoring System-EoEHSS) and molecular/genetic profile (EoE Diagnostic Panel—EDP score) at baseline at time of diagnosis and after treatment.

## Author Contributions

UK was responsible for the concept, research, drafting, and writing of this article.

### Conflict of Interest

The author declares that the research was conducted in the absence of any commercial or financial relationships that could be construed as a potential conflict of interest.
